# European guideline for imaging in paediatric and adolescent rhabdomyosarcoma — joint statement by the European Paediatric Soft Tissue Sarcoma Study Group, the Cooperative Weichteilsarkom Studiengruppe and the Oncology Task Force of the European Society of Paediatric Radiology

**DOI:** 10.1007/s00247-021-05081-0

**Published:** 2021-06-17

**Authors:** Roelof van Ewijk, Reineke A. Schoot, Monika Sparber-Sauer, Simone A. J. ter Horst, Nina Jehanno, Lise Borgwardt, Bart de Keizer, Johannes H. M. Merks, Alberto de Luca, Kieran McHugh, Thekla von Kalle, Jürgen F. Schäfer, Rick R. van Rijn, On behalf of the Cooperative Weichteilsarkom Studiengruppe Imaging Group, the European Society of Paediatric Radiology Oncology Task Force and the European Paediatric Soft Tissue Sarcoma Study Group Imaging Committee, Amine Bouhamama, Ana Coma, Pier Luigi Di Paolo, Raquel Davila Fajardo, Christiane Franzius, Chiara Giraudo, Gideon M. de Jonge, Daniel Levine, David MacVicar, Henry Mandeville, Shruti Moholkar, Carlo Morosi, Lil-Sophie Ording Müller, Erika Pace, Timothy N. Rogers, Sheila Terwisscha van Scheltinga, Nelleke Tolboom

**Affiliations:** 1 Princess Máxima Centre for Paediatric Oncology, Heidelberglaan 25, 3584 CS Utrecht, The Netherlands; 2 Paediatric Oncology, Olgahospital, Zentrum für Kinder-, Jugend- und Frauenmedizin, Klinikum Stuttgart, Stuttgart, Germany; 3 Department of Radiology and Nuclear Medicine, Wilhelmina Children’s Hospital University Medical Centre Utrecht, Utrecht, The Netherlands; 4 Department of Nuclear Medicine, Institut Curie, Paris, France; 5 Department of Clinical Physiology, Nuclear Medicine and PET, Rigshospitalet, Copenhagen, Denmark; 6 Department of Neurology, Brain Centre Rudolf Magnus, UMC Utrecht, Utrecht, the Netherlands; 7 Image Sciences Institute, UMC Utrecht, Utrecht, the Netherlands; 8 Department of Radiology, Great Ormond Street Hospital for Children, London, UK; 9 Institute of Radiology Olgahospital, Zentrum für Kinder-, Jugend- und Frauenmedizin, Klinikum Stuttgart, Stuttgart, Germany; 10 Division of Paediatric Radiology, Department of Radiology, University Hospital of Tübingen, Tübingen, Germany; 11 Department of Radiology and Nuclear Medicine, Amsterdam UMC, University of Amsterdam, Amsterdam, the Netherlands

**Keywords:** Adolescent, Child, Consensus, Diagnosis, Magnetic resonance imaging, Positron emission tomography/computed tomography, Rhabdomyosarcoma, Sarcoma, Staging

## Abstract

Appropriate imaging is essential in the treatment of children and adolescents with rhabdomyosarcoma. For adequate stratification and optimal individualised local treatment utilising surgery and radiotherapy, high-quality imaging is crucial. The paediatric radiologist, therefore, is an essential member of the multi-disciplinary team providing clinical care and research. This manuscript presents the European rhabdomyosarcoma imaging guideline, based on the recently developed guideline of the European Paediatric Soft Tissue Sarcoma Study Group (EpSSG) Imaging Committee. This guideline was developed in collaboration between the EpSSG Imaging Committee, the Cooperative Weichteilsarkom Studiengruppe (CWS) Imaging Group, and the Oncology Task Force of the European Society of Paediatric Radiology (ESPR). MRI is recommended, at diagnosis and follow-up, for the evaluation of the primary tumour and its relationship to surrounding tissues, including assessment of neurovascular structures and loco-regional lymphadenopathy. Chest CT along with [F-18]2-fluoro-2-deoxyglucose (FDG) positron emission tomography (PET)/CT or PET/MRI are recommended for the detection and evaluation of loco-regional and distant metastatic disease. Guidance on the estimation of treatment response, optimal long-term follow-up, technical imaging settings and standardised reporting are described. This European imaging guideline outlines the recommendations for imaging in children and adolescents with rhabdomyosarcoma, with the aim to harmonise imaging and to advance patient care.

**Supplementary Information:**

The online version contains supplementary material available at 10.1007/s00247-021-05081-0.

## Introduction

Medical imaging has a primary role in the care of children and adolescents with rhabdomyosarcoma, the most common soft-tissue sarcoma in childhood [1, 2]. In fact, it is essential for staging, response assessment, planning of local therapy (radiotherapy, surgery), monitoring for recurrence, and diagnosing acute complications and late sequelae of treatment. However, the optimal imaging approach has not been fully defined, resulting in a wide variation in clinical practice [3, 4].

The appropriate choice of imaging modality, protocols and timing of each assessment should be optimised to provide all information needed to guide therapy and follow-up for each child. An imaging guideline should lead to optimal collection of essential information, in balance with induced stress, sequelae and costs, taking into account the child’s age and developmental status [4], the psychosocial impact of investigations for the child and family [5, 6], and the physical impacts of radiation exposure and those of supporting measures like sequential sedation [4].

The treatment for paediatric patients with rhabdomyosarcoma is based on a multimodality approach: (multidrug) chemotherapy in combination with surgery or radiotherapy, in (very) high-risk patient categories followed by maintenance chemotherapy. Risk-group-adapted treatment is stratified on histology, PAX-FOXO1 status, Intergroup Rhabdomyosarcoma Study (IRS) post-surgical stage, age, tumour site, tumour size and regional nodal involvement. With this approach, 5-year overall survival is approximately 75% for people with localised disease and 30% for those with metastatic disease [7–12]. Because survival remains unsatisfactory, new trials are ongoing, such as the recently opened international Frontline and Relapsed Rhabdomyosarcoma (FaR-RMS) study (ClinicalTrials.gov Identifier: NCT04625907).

Recently, the European Paediatric Soft Tissue Sarcoma Study Group (EpSSG) Imaging Committee developed a guideline for imaging in children and adolescents with a rhabdomyosarcoma. EpSSG joined forces with the European Society of Paediatric Radiology (ESPR) Oncology Task Force and the Cooperative Weichteilsarkom Studiengruppe (CWS) Imaging Group to improve guideline quality, implementation and adherence and to facilitate data harmonization. We provide a review of available evidence followed by our imaging modalities recommendations for disease staging, response assessment and follow-up. We include technical guidance and standard report templates to improve standardisation and harmonisation of data.

The EpSSG Imaging Committee is composed of 22 paediatric radiologists/nuclear medicine physicians, one paediatric oncologist and 2 paediatric oncology clinical research fellows with expertise in paediatric sarcoma, representing 7 European countries. For evaluation and comparison of examinations within international multicentre studies and among collaborative groups, uniform methodology is essential. This urged us to develop a European rhabdomyosarcoma imaging guideline in collaboration with lead paediatric oncologists and paediatric radiologists of the EpSSG Imaging Committee, the CWS Imaging Group, the ESPR Oncology Task Force, paediatric surgeons and paediatric radiation oncologists.

## Diagnosis

### General

We refer to pictorial reviews on specific imaging characteristics of rhabdomyosarcoma and soft-tissue sarcoma to guide the differential diagnosis [13–16]. In the case of a suspected soft-tissue sarcoma, we recommend performing MRI of the primary tumour to guide the differential diagnosis. Planning and evaluation of all imaging should preferably be done by a paediatric radiologist and particularly in terms of hybrid imaging by a nuclear medicine physician with expertise in paediatric oncology. A full imaging workup should be performed before an imaging-guided core-needle or incisional biopsy, in collaboration with a paediatric surgeon, is obtained because the biopsy tract has to be included in any subsequent resection [17]. We recommend the use of technical imaging guidance and protocols (Online Supplementary Material 1). We also recommend the use of standardised templates for imaging reports (Online Supplementary Material 2).

### Primary tumour

Magnetic resonance imaging provides the most detailed characterisation of the tumour and surrounding tissues. The field of view of the MRI should include loco-regional lymph nodes and possible loco-regional extension of disease. Technically, we recommend performing an MRI including conventional sequences (T1, T2, post-contrast) in combination with diffusion-weighted imaging (DWI) MRI sequences. We refer to the technical section in this manuscript for detailed recommendations on specific MRI sequences as well as the essential use of fat suppression and apparent diffusion coefficient (ADC) maps. CT should not be used for primary evaluation of the tumour and is only occasionally useful for additional assessment of subtle bone erosion or destruction, for example in rhabdomyosarcoma near the skull base. We recommend performing one-dimensional (1-D) measurements according to RECIST (response evaluation criteria in solid tumors) [18, 19]. Optionally, three-dimensional (3-D) assessment can be performed [20]. The MRI report should describe the primary tumour including its size, characteristics, local extension and relation to the surrounding anatomical structures.

We highly recommend pre-treatment re-evaluation of the primary tumour in case of an excisional biopsy or if an MRI has been performed more than 4 weeks before start of therapy.

### Regional lymph nodes

Regional nodal staging is important because tumour-positive lymph nodes are an independent poor prognostic factor for survival in rhabdomyosarcoma, with impact on treatment stratification and need for radiotherapy of involved nodes. Regional nodal disease (N1) is present in 23% of all people with rhabdomyosarcoma [21–24]. Pathological non-regional lymph nodes should be identified as metastatic disease and treated accordingly.

Magnetic resonance imaging should be combined with [F-18]2-fluoro-2-deoxyglucose (FDG) positron emission tomography (PET)/CT or PET/MRI for evaluating loco-regional lymph nodes. Regional lymph nodes are defined as those draining the site of the primary tumour. Table 1 shows an overview of the most common tumour sites with their corresponding loco-regional lymph nodes. However, different lymph node pathways exist. It is, therefore, important to include a complete assessment of (potential) regional lymph nodes, even if nearby lymph nodes seem normal; this is based on research in people with sarcoma and melanoma [25–28]. In rhabdomyosarcoma localised in the upper extremity, the field of view covers the lymph nodes located in the axilla and the clavicular region, even if cubital/epitrochlear and axillary lymph nodes are normal. In rhabdomyosarcoma localised in the lower extremity, the field of view includes the pelvic lymph nodes, even if femoral and popliteal nodes are normal on US [29]. In para-testicular rhabdomyosarcoma, a full assessment includes the para-aortic, iliac and inguinal lymph nodes [30].

**Table 1:** Comprehensive guidance of the regional lymph nodes defined as those appropriate to the site of the primary tumour

Region	Definition
Extremities	
Upper — hand and forearm	Epitrochlear nodes along brachial vessels, deltopectoral nodes, axillary nodes
Upper — upper arm	Nodes along brachial vessels, deltopectoral nodes, axillary nodes
Upper — shoulder	Axillary nodes, subclavian nodes
Lower — foot and leg	Popliteal nodes, nodes along femoral vessels, inguinal nodes
Lower — thigh	Inguinal nodes, adductor region of the thigh, external iliac nodes
Lower — buttock	Inguinal nodes, hypogastric nodes (internal iliac nodes)
Genitourinary	
Bladder — prostate	Pelvic (hypogastric, obturator, iliac, perivesical, pelvic, sacral and presacral lymph nodes). Note: para-aortic nodes are distant nodes
Cervix	Pelvic (hypogastric, obturator, iliac, perivesical, pelvic, sacral and presacral lymph nodes). Note: para-aortic nodes are distant nodes
Uterus	Pelvic, retroperitoneal nodes at renal vessels or below
Paratesticular/gonadal	Ipsilateral pelvic, retroperitoneal nodes at renal vessels or below (inguinal if the scrotum is involved)
Vagina	Retroperitoneal, pelvic nodes at or below common iliac vessels, inguinal nodes
Vulva	Inguinal nodes
Head and neck	
Head/neck	Ipsilateral cervical, jugular, pre-auricular, occipital, supraclavicular nodes for laterally placed tumours (excluding scalp); may have bilateral lymphadenopathy if the tumour is central
Orbit/eyelid/cheek/ external ear/temporal region	Parotid, ipsilateral jugular, pre-auricular, cervical nodes
Trunk	
Intrathoracic	Internal mammary, mediastinal nodes
Chest wall	Axillary, internal mammary, infraclavicular nodes
Intra-abdominal	Sub-diaphragmatic, peritoneal, mesenteric and iliac lymph nodes according to site
Abdominal wall	Inguinal, femoral nodes
Retroperitoneum/pelvis	Pelvic and retroperitoneal nodes
Other	
Biliary/liver	Porta hepatis nodes
Perianal, perineal	Inguinal, pelvic nodes; may be bilateral

Regional nodal assessment should be performed with MRI, in line with RECIST 1.1, in combination with FDG PET/CT or PET/MRI. Pathological lymph nodes must meet the criterion of a short axis of ≥15 mm; lymph nodes with a short axis <10 mm are considered non-pathological [18, 19]. Although RECIST 1.1 guidelines have been developed for adult oncological patients, recent studies in healthy children and adolescents justify the use of these cut-offs with the exception of cervical lymph nodes [31, 32]. In fact, apart from the upper internal jugular nodes (level II), a short-axis diameter exceeding 10 mm is uncommon in healthy children [31]. Any FDG-positive lymph node with a short axis <15 mm should be considered suspicious, with FDG positivity defined as uptake greater than normal liver FDG uptake without a known physiological explanation [18, 19, 33]. We strongly recommend performing a biopsy in the case of doubtful nodal involvement based on imaging characteristics (abnormal node morphology), short axis >10 mm or equivocal FDG uptake.

Children with rhabdomyosarcoma of the extremities [29, 34, 35] and people age 10 years and older with paratesticular rhabdomyosarcoma [36–39] should receive surgical staging of nodes irrespective of imaging results. This is strongly recommended, in line with current EpSSG and International Soft-tissue Sarcoma Database Consortium (INSTRuCT) surgery guidelines, because some clinically and radiologically normal-appearing lymph nodes show tumour cells on histological examination [29, 36, 38, 39].

### Metastatic disease

Approximately 15–20% of children with rhabdomyosarcoma initially present with metastatic disease; this is related to a poor prognosis, with an average 5-year survival of 30% [9, 12, 40]. Common sites for metastatic disease are the lungs (47%), bone marrow (38%), bone (34%) and distant lymph nodes (26%) [9]. Among people with pulmonary metastatic disease, 29% have combined metastatic disease to other sites [9, 40].

For the optimal detection of distant metastatic disease, we recommend FDG PET/CT or PET/MRI in combination with a diagnostic chest CT, acquired on maximum inspiration to exclude lung nodules [41, 42]. For evaluation of lung nodules, lung MRI can be an alternative to chest CT, but only in appropriate settings and with sufficient experience [43, 44]. Whole-body FDG PET/CT or PET/MRI, head to toe, is mandatory because metastases can be asymptomatic. Identification of all lesions is essential for optimal local treatment of metastatic disease [45]. In the future, whole-body MRI with diffusion-weighted MRI sequences might become a radiation-free alternative to FDG PET. Prospective clinical studies are warranted to validate its role in staging [46].

FDG PET/CT or PET/MRI is recommended for evaluating nodal and distant metastases because studies show a higher sensitivity and specificity to identify nodal disease when compared with conventional imaging as well as superior detection of bone and bone marrow metastases than with technetium-99 m bone scintigraphy. A systematic review of FDG PET imaging reported a sensitivity of 80–100% and a specificity of 89–100% at detecting nodal involvement [33, 47]. For distant metastatic non-pulmonary involvement, the sensitivity of FDG PET ranged from 95% to 100% and the specificity from 80% to 100% [47, 48]. Technically, an FDG PET/CT should be performed according to the current European Association of Nuclear Medicine (EANM) guidelines [49, 50]. For clinical interpretation of FDG PET/CT or PET/MRI, we recommend performing visual assessment and considering lymph nodes suspicious for malignancy if FDG uptake is greater than normal adjacent background tissue without a known physiological explanation, using a Deauville-like score where liver uptake is used as reference tissue. Although recommended, visual assessment according to the Deauville criteria (Table 2), normally used in malignant lymphoma response assessment [51], has not been systematically evaluated in staging nodal disease in paediatric soft-tissue sarcoma. For response assessment in clinical studies, we recommend additional quantitative measurements using standardised uptake values (SUV) according to PERCIST (PET response criteria in solid tumors) [52, 53].

**Table 2:** [F-18]2-fluoro-2-deoxyglucose (FDG) positron emission tomography (PET) visual scoring system: Deauville-like 5-point scale

Score	Definition
1	No uptake
2	Uptake ≤ mediastinum
3	Uptake > mediastinum but ≤ liver
4	Uptake moderately more than liver uptake, at any site
5	Markedly increased uptake at any site and/or new sites of disease

Because small pulmonary metastatic lesions can be missed on FDG PET/CT, a CT of the lungs of diagnostic quality should either be part of the FDG PET/CT or performed separately [47, 54]. Sub-centimetre pulmonary nodules are frequently identified in children with rhabdomyosarcoma and in otherwise healthy children [55]. The EpSSG RMS2005 study demonstrated that people with small pulmonary nodules (≤ four nodules <5 mm, or one nodule measuring ≥5 mm to <10 mm) have a similar survival as those with localised disease [56]. Histopathological examination, which was considered the gold standard for final characterisation of these nodules, is not recommended because it does not change the treatment for the patient or improve survival. Therefore, we recommend classifying “indeterminate nodules” if four or fewer nodules smaller than 5 mm or one nodule measuring 5–10 mm is identified. In the absence of other metastases, these patients should be treated as having local disease. This classification should be incorporated in the conclusion of the chest CT report.

## Response assessment in upfront treatment

### Timing

Current EpSSG and CWS protocols use multidrug chemotherapy, most often in 3-week cycles. Induction chemotherapy in previous and upcoming EpSSG trial protocols consists of 9 three-weekly cycles with radiotherapy and/or surgery after course 4 (in patients with localised disease) or course 6 (in patients with metastatic disease) when indicated. In the upcoming FaR-RMS trial, high-risk and very-high-risk patients are being randomised to maintenance therapy lasting either 6 months or 12 months, or 12 months or 24 months, respectively.

We recommend unifying early response assessment and assessing the initial response after 2–3 courses (reflecting 6–9 weeks) of chemotherapy, in line with current common practice. Furthermore, we suggest re-assessing the disease status before local therapy in case of distant metastatic disease (after course 6), at end of induction chemotherapy, after every 6 cycles of maintenance therapy and at completion of therapy (Table 3).

**Table 3:** Summary of recommendations for imaging in paediatric rhabdomyosarcoma

	Induction therapy Cycles 1–9			Maintenance therapy HR: 6 or 12 cycles VHR: 12 or 24 cycles
	Staging	During	End of induction	
Imaging of the tumour site(s)^a^	•	After cycles 3, 9 (after 6 in case of distant metastatic disease — very high risk group)		HR: After cycles 6, 12 VHR: After cycles 6, 12, 18, 24
Chest CT^b^	•	If positive after 3 cycles, repeat after cycle 6	If positive at staging, repeat at end of induction treatment	As clinically indicated
FDG PET/CT or PET/MRI^c^	•	As per local practice After cycle 3 for HR/VHR patients in FDG PET substudy Recommended to repeat in case of FDG PET positive lymph nodes or FDG PET positive distant metastases at diagnosis until negative		

*FDG PET* [F-18]2-fluoro-2-deoxyglucose positron emission tomography, *HR* high risk, *VHR* very high risk

^a^MRI is recommended for all anatomical regions

^b^Repeat chest CT is recommended only if there is pulmonary involvement at baseline

^c^FDG PET/CT or PET/MRI is the investigation of choice; otherwise, as per local practice. Use same mode of investigation throughout the study. Children with FDG-PET-positive lymph nodes or FDG-PET-positive distant metastases at diagnosis are recommended to have repeat FDG PET scans until negative (or in case of another explanation of persisting FDG PET avidity, e.g., post irradiation)

### Primary tumour

In previous European treatment protocols, 3-D measurements were used for tumour size response assessment and treatment was adjusted in case of insufficient response or progressive disease [57]. Six studies analysed the prognostic value of tumour size response, measured either in one (RECIST criteria), two (World Health Organization methodology) or three dimensions (EpSSG/CWS methodology). Four studies found no prognostic value for tumour size response [58–61]. In two positive prognostic studies, people with progressive disease at early response measurements were included, potentially explaining the differences in outcomes between studies [62, 63]. In conclusion, studies do not show a clear difference in survival among stable disease, partial response and complete response in children and adolescents with rhabdomyosarcoma. Progressive disease is known to correlate with a poor survival, as compared to any other response [58–63]. Current protocols, therefore, only switch to second-line therapy in case of confirmed progressive disease. Progressive disease is defined as an increase of target lesions in one dimension of 20% or more as measured, conforming with the RECIST 1.1 guidance. In case of multiple target lesions, progressive disease is defined as 20% or more increase for the sum of longest diameters of the target lesions [18, 19]. If response of the primary tumour is assessed in three dimensions, as per previous EpSSG protocols, a volume increase of 73% correlates with a 20% 1-D increase [20]. Progressive disease can also be diagnosed in case of the occurrence of new lesions or enlargement of non-target lesions [18, 19].

### Metastatic disease

Although in metastatic rhabdomyosarcoma limited data show no value of early tumour size response of the primary tumour, potential prognostic value of complete response of lung metastases is reported [64]. Metastatic lesions, which qualify as target lesions, should be measured in one dimension, conforming with the RECIST 1.1 guidance [18, 19]. In case of pulmonary metastatic disease at diagnosis, chest CT should be repeated every three courses during induction chemotherapy to evaluate the response of all target lesions, according to RECIST 1.1 criteria. FDG PET/CT or PET/MR imaging is encouraged to assess the value of this modality in both identifying sites of disease and measuring tumour response. Children with FDG-avid lymph nodes or FDG-avid distant metastases, with a Deauville score 4–5 at diagnosis, are recommended to have repeat PET scans after every three cycles until negative (FDG uptake in previously positive nodes is decreased to activity in or below normal liver parenchyma, as visually read based on Deauville criteria). FDG PET response should be assessed according to PERCIST [52, 53] in the research setting. In general, children with FDG-negative lymph nodes or metastases do not need further FDG PET/CT or PET/MR imaging. In the FaR-RMS trial, FDG PET/CT or PET/MRI response is an official sub-study to determine whether this is a prognostic biomarker for local failure and survival and is, therefore, routinely performed after three cycles of chemotherapy.

### Response measurement variability

One-dimensional measurements as defined in RECIST 1.1 are common practice, although this has not been validated in children [18, 19]. Independent of the chosen method, either 1-D according to RECIST 1.1 or 3-D measurements should be carefully performed and interpreted because interobserver and inter-method variability can unfortunately lead to different response classifications, with treatment consequences in a considerable proportion of patients [20]. Because both methods show equal variability, we recommend measuring the longest dimension and all three diameters on the same pulse MR sequence (ideally with all measurements by the same expert). For consistency and standardisation of response assessment, we strongly advocate the use of blinded or central review for the classification of progressive disease.

### Quantitative imaging response markers

Neither imaging nor biological markers reflect the degree of tumour response to chemotherapy or indicate therapy’s effect on prognosis. Upcoming imaging modalities to assess tumour response are FDG PET/CT or PET/MRI, diffusion-weighted MRI (DWI) and dynamic contrast-enhanced MRI. FDG PET, classified according to the PERCIST criteria or quantified in absolute standardised uptake value (SUV), is promising but has not been validated in large cohorts. One study consisting of 23 people with rhabdomyosarcoma showed, in a subset of 13 patients, FDG-PET-assessed complete metabolic response in 69% of patients compared with 8% with a conventional imaging methodology [48, 65]. One study consisting of 107 patients, both with localised and metastatic disease, showed complete metabolic response in 45 patients after 12 weeks of induction chemotherapy [66]. Response to therapy correlated with survival, reflected by a 3-year progression-free survival of 72% (95% confidence interval [CI] 58–86%) in patients with negative FDG PET scans, versus a 3-year progression-free survival of 44% (95% CI 31–57%) in those with positive FDG PET scans (*P*=0.01) [66]. DWI and dynamic contrast-enhanced MRI sequences provide indirect characterisation of the cellularity and vascularisation of the tumour, respectively. To date, DWI is considered the most promising technique for evaluating tumour response to chemotherapy. DWI measures the average displacement of water molecules, which can be used as an indirect marker to investigate the microstructure of tissues. In malignant tumours, characterised by high cellularity, water diffusion is thought to be hindered and restricted by tightly packed cellular membranes. A decrease in cellularity as a result of treatment response has been linked to an increase in water diffusion [67]. Quantitative DWI indices, such as the apparent diffusion coefficient (ADC), have been shown to be potential prognostic markers in different cancers [68–71]. However, for rhabdomyosarcoma only limited data have been published [72]. The combination of the metabolic information (e.g., SUV) and quantitative DWI could be an interesting treatment response measurement. Future studies need to define whether qualitative or quantitative assessment results in validated markers of early tumour response. Identifying an imaging biomarker of early response, associated with survival, could improve the evaluation of new therapies. For example, defining effective clinical endpoints in phase I/II trials for rhabdomyosarcoma will lead to faster selection of effective agents and regimens for the patients. Moreover, this will support drug approval by legal authorities. For future purposes and multi-centre analyses, we strongly advocate performing DWI, including ADC maps, according to standardised protocols in children and adolescents with rhabdomyosarcoma.

### Recommended response evaluation

We recommend MRI of the primary tumour for evaluating response to chemotherapy, FDG PET/CT or PET/MRI for evaluating nodal and metastatic disease, and chest CT for evaluating lung metastases. Response is primarily measured one-dimensionally (according to RECIST 1.1 guidance). Three-dimensional volumetric assessment is an alternative methodology. Progressive disease is a 20% 1-D increase according to RECIST 1.1, which corresponds with a 73% increase in 3-D assessment, or the appearance of new lesions. Progressive disease is the only indication for adjusting to second-line therapy. Radiologists and nuclear medicine physicians should be aware of interobserver variability when performing tumour measurements. New potential markers of response from FDG PET/CT or PET/MRI, and DWI could become prognostic biomarkers; however, limited data have been published. We strongly advocate adherence to standardised MRI protocols and blinded/central review, particularly when progressive disease is suspected.

## Response assessment in case of residual lesions at the end of therapy

All patients receive an end-of-treatment evaluation including MRI of the primary tumour and a chest CT in case of pulmonary metastatic disease at diagnosis, otherwise a chest radiograph. Previous research has demonstrated that up to 35% of people with rhabdomyosarcoma who start with gross residual disease (IRS III group) after initial biopsy continue to have some sort of residual disease at the end of therapy [73].

In two recent trials, the Children’s Oncology Group (COG) analysed the prognostic effect of end-of-therapy radiologic response. In non-parameningeal rhabdomyosarcoma, failure-free survival was not different for children with no response/partial response or without macroscopic residual disease on imaging. In parameningeal rhabdomyosarcoma, failure-free survival was higher, with a 5-year failure-free survival of 74.5% (95% CI 66.5–82.6%) in cases of complete response compared to 62.7% (95% CI 52.4–73.0%) in children with partial response/no response (*P*=0.038). However, overall survival was not significantly different in these groups of children [74].

Although no specific MRI characteristics of the residual lesion have been described to suggest a higher risk for relapse, studies that retrospectively investigated end-of-therapy FDG PET showed a predictive value of FDG PET response [47, 66]. FDG PET response after induction chemotherapy is under investigation in the COG rhabdomyosarcoma protocol [74] and the EpSSG FaR-RMS study. We underline the need for a study investigating the value of an end-of-therapy FDG PET scan in IRS III/IV patients with residual disease.

## Imaging standard for radiotherapy

Radiotherapy is an essential part of the treatment of paediatric and adolescent rhabdomyosarcoma [75]. Imaging of the primary tumour and its metastatic sites is essential to define the optimal radiotherapy plan. All relevant diagnostic imaging and response assessment cross-sectional imaging, including FDG PET/MRI or CT/MRI, are recommended as discussed in this guideline, following the timing and technical guidance provided. Of specific notice, in cases of metastatic disease, we recommend repeat imaging after six cycles of chemotherapy, before the start of local therapy. In cases of severe metastatic disease, where irradiation of all metastatic lesions is not feasible, an FDG PET/CT or PET/MRI after chemotherapy can guide towards irradiation of persistent metabolically active sites, although no evidence supports this approach. Radiotherapy planning CT or MRI is performed according to local expertise because there are no general guidelines on this specific topic.

## Off-therapy follow-up imaging

The common hypothesis is that early detection of a tumour by imaging leads to improved prognosis. Two studies retrospectively investigated the value of early relapse detection, including 271 patients, mainly with localised disease [76, 77]. More than half of the patients with a relapse presented with clinical symptoms, most often a palpable mass or pain; the asymptomatic patients whose recurrence was detected by imaging did not show an improved survival [76, 77]. Unfortunately, the numbers did not allow for tumour-site-specific sub-analyses. The authors estimated that 178 MR scans of the primary site and 178 chest radiographs need to be performed to identify 1 patient with an asymptomatic relapse [76]. Although these studies are retrospective and should be interpreted with caution, the effect of sedation in young children with potential long-term effects [78], the increased radiation exposure and the induced stress for the children and their families from the investigation [5, 6] should be balanced to the suspected benefit of long-term follow-up imaging and risk for relapse.

In localised rhabdomyosarcoma the risk for recurrence depends on the known risk factors and correlates with event-free survival. The median time to relapse differs per study between 8 months and 1.5 years, ranging from several months to more than 10 years. Most relapses occur in the first 2 years post therapy [40, 57, 79]. In children with systemic relapses, lung metastases are most often identified [76, 80]. In metastatic rhabdomyosarcoma survival is generally poor, with a 5-year event-free survival between 20% and 30% for the whole group. In the case of two or more Oberlin risk factors being present, the 5-year event-free survival is 12% as compared to 40% in the case of 1 or 0 Oberlin risk factors [40]. Chances for survival after relapse in children with metastatic disease at first diagnosis are dismal.

Even though the value of follow-up imaging is questioned, no study has been performed prospectively or randomised. Therefore, following completion of treatment we recommend performing MR examination of the primary tumour (in case of superficial lesions, US could be an alternative) and screening for lung metastases through chest radiograph for the first 2 years. We suggest a total of six assessments, every 4 months. In case of a suspicious nodule on radiography, CT is advised for characterisation. After 2 years, we recommend imaging only if new clinical symptoms develop because routine screening has limited added detection value.

### Cancer predisposition syndromes

In rare cases, children with a rhabdomyosarcoma have an underlying tumour predisposition syndrome such as Li–Fraumeni, DICER1, Gorlin, neurofibromatosis type 1, Beckwith–Wiedemann, Costello, Noonan or Rubinstein–Taybi syndrome or, in some, reported deoxyribonucleic acid (DNA)-repair defects [81, 82]. For children with a cancer predisposition syndrome, we refer to specific cancer surveillance guidelines as published, for example, in Li–Fraumeni [83] and DICER1 [84] syndromes, for individualised screening [46].

### Relapse staging and response

In the case of recurrence, we recommend performing full staging and later response in line with the imaging evaluations defined in this manuscript.

### Imaging sequelae

Rhabdomyosarcoma is the most common sarcoma in children. Site-specific sequelae from chemotherapy and radiotherapy need specific attention in a developing child. For example, in head and neck rhabdomyosarcoma, dental growth deficits and asymmetrical development of facial bones have been described [85–89]. We did not define specific guidance for evaluating sequelae.

## Technical recommendations

### Chest computed tomography

The ideal slice thickness of a CT scan is 1.0–1.5 mm. The examination should be performed in full inspiration, wherein supine position and instructions, if possible for the child, are advised. Reading should be performed at minimum in axial and coronal planes; the use of maximum-intensity projections is advised to improve sensitivity [55, 90, 91].

### Magnetic resonance imaging

Magnetic resonance imaging has superior soft-tissue contrast resolution and absence of ionising radiation, allowing for detailed characterisation. An optimal MR protocol should lead to collection of all essential information in balance with the known costs, the induced stress, and the long-term effects. We propose that this imaging protocol, designed for both 1.5-tesla (T) and 3.0-T scanners for the three most common vendors (Siemens Healthcare, Philips Healthcare and GE Healthcare), becomes the minimal standard. Because rhabdomyosarcoma can present anywhere in the body, we developed specific acquisition protocols for the head and neck region, the chest and abdominal region, and the extremities. We strongly recommend implementing and adhering to the technical settings (orientation, slice thickness, voxel size, echo time, repetition time, b values) provided in Online Supplementary Material 1 and encourage the use of standardised reports (Online Supplementary Material 2).

Both 1.5 T and 3.0 T are suitable for visualising paediatric rhabdomyosarcoma. We recommend performing imaging on the best available local machine, wherein updates on hardware, software and available fitted coils are accounted for. In general, 3.0 T is preferred for rhabdomyosarcoma located in the head and neck region and in the extremities. For rhabdomyosarcoma in the chest or abdomen, 1.5 T is preferred. The proper choice and adjustment of surface coils is essential, especially in small tumours. The core of the MR protocol consists of morphological sequences (coronal and axial T1- and T2-weighted, including a fat-suppressed T2) with the administration of contrast agent, for example gadolinium (post-contrast coronal and axial T1-W post-gadolinium). DWI sequences add to characterisation of the tumour and tumour response. We strongly advise using a minimum of four b values (0, 100, 500 and 1,000 s/mm^2^) with at least three orthogonal directions, but preferably more than six. The b values have been defined as part of the EpSSG RMS Imaging Guideline to aim for optimal harmonisation of imaging. To this moment, no systematic evaluation of the optimal number of b values in rhabdomyosarcoma has been performed. As such, for visual evaluation of diffusion MR, an absolute minimum of two b values (0 and 1,000) is required. We consider dynamic contrast-enhanced MRI not as standard, but as optional to local practice and expertise. It is important to save all files in a Digital Imaging and Communications in Medicine (DICOM) format, including the raw DWI data, for future potential harmonisation or re-processing with up-to-date algorithms.

### [F-18]2-fluoro-2-deoxyglucose (FDG) positron emission tomography (PET)

We recommend performing FDG PET/CT in line with current EANM guidelines [49, 50], or FDG PET/MRI, wherefore there are no guidelines, only recommendations [92–94]. It is important to image the whole body, including the skull and the extremities, because silent lesions can be missed based on clinical symptoms [41].

## Future and research perspective

The engagement of paediatric radiologists and nuclear medicine physicians is important to improve imaging and treatment strategies in children with rhabdomyosarcoma. Trials investigating new treatment strategies depend on repeated assessments with radiologic examinations, often including imaging endpoints. An imaging guideline is also expected to improve research in paediatric and adolescent rhabdomyosarcoma. In Europe, and across the globe, we must strive for optimal care for children with rhabdomyosarcoma by well-performed high-quality imaging. Therefore, engaging with the European paediatric radiology community is essential for implementing and adhering to this guideline. Where previously radiologists were only partly involved in protocol writing, we have seen a change. It has been shown that performing collaborative imaging research in rhabdomyosarcoma leads to high-impact publications with important clinical implications for these children [20, 46, 56, 61, 76]. We strongly encourage further collaborative imaging studies in rhabdomyosarcoma.

We foresee new research projects to investigate and determine the optimal role of FDG PET/CT and PET/MRI, DWI and whole-body MRI in staging and response assessment in rhabdomyosarcoma. Currently there are no early markers of response with the accuracy needed to predict outcome. Therefore, clinical trials now last up to 7–10 years before we know whether a new strategy leads to improved survival [57]. Finding and validating imaging biomarkers of poor versus good response could result in faster selection of (non)promising agents and treatment strategies. Moreover, by detecting good responders to chemotherapy, we might identify children wherein therapy might be reduced, which has been shown in paediatric Hodgkin lymphoma [95–97]. Imaging could thereby help to lower the burden of therapy. Also, imaging research itself should aim to minimise the burden of repetitive examinations by defining optimal modalities and optimal response time-points, evaluating the potential of risk-adapted staging and identifying risk-groups where follow-up imaging adds to survival. For this, we need to prospectively validate findings from retrospective studies, for example of the published studies on follow-up imaging [76].

What is needed to perform further studies? We will only be able to perform large reliable validating studies by: (1) collaborating and extending the network of paediatric radiologists and nuclear medicine physicians actively involved in imaging research; (2) actively engaging in and sharing research interests; (3) creating willingness to tailor and harmonise protocols; (4) sharing data for central review and connecting with imaging researchers; (5) providing a platform suitable for systematic pseudonymised collection of standardised imaging data as part of prospective clinical trials; and (6) connecting the imaging data with clinically relevant output, to explore radiomics and methods like machine learning.

## Conclusion

This manuscript illustrates the European guideline for imaging of rhabdomyosarcoma in children and adolescents, on behalf of the EpSSG Imaging Committee, the CWS Imaging Group and the Oncology Task Force of the ESPR. Optimal imaging is an essential part of the multidisciplinary clinical care and research in rhabdomyosarcoma, in which experts in paediatric oncology imaging play a crucial role. Implementation and adherence to technical recommendations, adequate modalities, timing of the examinations, response assessment criteria and the standard reporting templates provided should lead to international harmonisation and to improved care for children with rhabdomyosarcoma.

## Supplementary Information

Online Supplementary Material 1 (DOCX 47 kb)

Online Supplementary Material 2 (DOCX 15 kb)
