# Supplementary material for: European guideline for imaging in paediatric and adolescent rhabdomyosarcoma — joint statement by the European Paediatric Soft Tissue Sarcoma Study Group, the Cooperative Weichteilsarkom Studiengruppe and the Oncology Task Force of the European Society of Paediatric Radiology
Source: Pediatr Radiol. 2021 Jun 17;51(10):1940–51. doi: 10.1007/s00247-021-05081-0 (PMC8426307; doi:10.1007/s00247-021-05081-0)
Supplement: Supplementary file 1 — (DOCX 47 kb) [file 247_2021_5081_MOESM1_ESM.docx]

## Appendix A

## Technical imaging guidance and protocols

## Philips 3-tesla (T) rhabdomyosarcoma MR template

**Head and neck region**

| **INDICATION** | | | | | | **PROTOCOL** | | | | |
| --- | --- | --- | --- | --- | --- | --- | --- | --- | --- | --- |
| Soft-tissue tumour in the head or neck | | | | | | Before contrast agent administration: sequences 1 to 6 | | | | |
|  | | | | | | DCE optional | | | | |
| If possible mark swelling or scar | | | | | | | | | | |
|  | | | | | | | | | | |
| **PREPARATION** | | | | | | | | | | |
| Coil | | Head / neck | | | | | | | | |
| Contrast agent | | Gadolinium (for instance, Gadovist 0,1 mL per kg body weight) | | | | | | | | |
|  | | | | | | | | | | |
|  | Sequence | | Technique | Orientation | FOV [mm] | Slices | Thickness [mm] | Voxel size [mm] | TE [ms] | TR [ms] |
| **1** | SURVEY | | TFE | MST |  |  |  |  |  |  |
| **2** | T1 TSE | | TSE | COR | 180 x 180 | 33 | 3.0 | 0.45 x 0.5 | 15 | 450 / 700 |
| **3** | T1 TSE | | TSE | TRA | 180 x 180 | 35 | 3.0 | 0.6 x 0.66 | shortest 15 | 450 / 750 |
| **4** | T2 TSE | | TSE | COR | 180 x 180 | 33 | 3.0 | 0.5 x 0.5 | 80 | 2,500 / 6,000 |
| **5** | T2 Fat saturation (FS) | | MV | TRA | 330 x 330 | 64 | 3.0 | 0.8 x 0.8 | 96 | 3,000 / 4,500 |
| **6** | DWI (b=0; 100; 500; 1,000 s/mm2) and ADC map | | EPI 2-D | TRA | 230 x 196 | 41 | 3.0 | 2.0 x 2.0 | shortest 75 | 4,000-5,000 |
| **7** | Post-Gd eTHRIVE | | TFE | TRA | 190 x 190 | 320 | 0.9 | 0.5 x 0.8 | shortest 3.2 | shortest 6.6 |
| **8** | Post-Gd T1 TSE DIXON | | TSE | TRA | 200 x 200 | 35 | 3.0 | 0.6 x 0.78 | 14 | 450 / 650 |
| **9** | Post-Gd T1 TSE DIXON | | TSE | COR | 180 x 180 | 33 | 3.0 | 0.6 x 0.78 | 14 | 450 / 650 |

**Chest and abdomen**

1.5 T recommended, see 1.5-T protocol.

**Extremities**

| **INDICATION** | | | | **PROTOCOL** | | | | | | |
| --- | --- | --- | --- | --- | --- | --- | --- | --- | --- | --- |
| Soft-tissue tumour arising from the extremities | | | | Before contrast agent administration: sequences 1 to 5 | | | | | | |
|  | | | | During contrast agent administration: sequence 6 | | | | | | |
|  | | | | After contrast: sequence 7 and 8 with SPIR | | | | | | |
| If possible mark swelling or scar | | | |  | | | | | | |
|  | | | | | | | | | | |
| **PREPARATION** | | | | | | | | | | |
| Coil | | Posterior and anterior | | | | | | | | |
| Contrast agent | | Gadolinium (for instance, Gadovist 0.1 mL per kg body weight) | | | | | | | | |
|  | | | | | | | | | | |
|  | Sequence | | Technique | Orientation | FOV [mm] | Slices | Thickness [mm] | Voxel size [mm] | TE [ms] | TR [ms] |
| **1** | SURVEY | | FFE | MST |  |  |  |  |  |  |
| **2** | T1 TSE | | TSE | TRA | 250 x 250 | 30 | 4.0 | 0.8 x 1.1 | 10 | 400 / 750 |
| **3** | T2 TSE mDIXON | | TSE | TRA | 250 x 250 | 30 | 4.0 | 0.87 x 1.09 | 80 | shortest 2,236 |
| **4** | T1 | | TSE | SAG/COR | 200 x 200 | 40 | 3.0 | 0.8 x 0.9 | 10 | 500 / 700 |
| **5** | DWI (b=0; 100; 500; 1,000 s/mm2) and ADC map | | EPI | TRA | 230 x 230 | 50 | 4.0 | 2.5 x 2.5 | shortest 54 | 4,000-5,000 |
| **6** | DCE (start < 20 s after infusion for fast temporal resolution) | | TFE | TRA | 300 x 150 | 8 | 5..0 | 1.17 x 2.54 | 1..5 | shortest 6.1 |
| **7** | Post-Gd T1 TSE SPIR | | TSE | SAG/COR | 200 x 200 | 40 | 3..0 | 0.8 x 0.9 | 10 | 500 / 700 |
| **8** | Post-Gd T1 TSE SPIR | | TSE | TRA | 250 x 250 | 30 | 4..0 | 0.8 x 1.1 | shortest 10 | 400 / 750 |

## Philips 1.5-T rhabdomyosarcoma MR template

**Head and neck**

| **INDICATION** | | | | | | **PROTOCOL** | | | | |
| --- | --- | --- | --- | --- | --- | --- | --- | --- | --- | --- |
| Soft-tissue tumour in the head or neck | | | | | | Before contrast agent administration: sequences 1 to 6 | | | | |
|  | | | | | |  | | | | |
| If possible mark swelling or scar | | | | | | | | | | |
|  | | | | | | | | | | |
| **PREPARATION** | | | | | | | | | | |
| Coil | | Head / neck | | | | | | | | |
| Contrast agent | | Gadolinium (for instance, Gadovist 0.1 mL per kg body weight) | | | | | | | | |
|  | | | | | | | | | | |
|  | Sequence | | Technique | Orientation | FOV [mm] | Slices | Thickness [mm] | Voxel size [mm] | TE [ms] | TR [ms] |
| **1** | SURVEY | | FFE | MST |  |  |  |  |  |  |
| **2** | T1 TSE | | TSE | COR | 180 x 180 | 33 | 3.0 | 0.6 x 0.74 | 14 | 400 / 650 |
| **3** | T1 TSE | | TSE | TRA | 180 x 180 | 35 | 3.0 | 0.6 x 0.74 | 16 | 400 / 650 |
| **4** | T2 TSE | | TSE | COR | 180 x 180 | 33 | 3.0 | 0.45 x 0.5 | 100 | 2,500 / 3500 |
| **5** | T2 FS | | MV | TRA | 330 x 330 | 64 | 3.0 | 1.0 x 1.0 | shortest 76 | shortest 6063 |
| **6** | DWI (b=0; 100; 500; 1,000 s/mm2) and ADC map | | EPI | TRA | 200 x 200 | 41 | 4.0 | 2.81 x 2.81 | shortest 71 | 4,000-5,000 |
| **7** | Post-Gd eTHRIVE^#^ | | TFE | TRA | 190 x 190 | 320 | 1.0 | 0.8 x 0.9 | shortest 4.5 | shortest 9.6 |
| **8** | Post-Gd T1 TSE DIXON^#^ | | TSE | TRA | 180 x 180 | 35 | 3.0 | 0.7 x 0.92 | 14 | 400/ 700 |
| **9** | Post-Gd T1 TSE DIXON^#^ | | TSE | COR | 180 x 180 | 33 | 3.0 | 0.7 x 0.94 | 14 | 400 /700 |

^#^ Post-Gd scans should be performed with fat saturation / water excitation.

**Chest and abdomen**

| **INDICATION** | | | | | | | **PROTOCOL** | | | | | |
| --- | --- | --- | --- | --- | --- | --- | --- | --- | --- | --- | --- | --- |
| Soft-tissue tumour in chest or abdomen | | | | | | | Before contrast agent administration: sequences 1 to 11 | | | | | |
|  | | | | | | | After contrast agent administration: repeat sequence 3 and 4 (=sequence 12 and 13) | | | | | |
|  | | | | | | |  | | | | | |
|  | | | | | | | Sequence 7 to 11 set around the mass | | | | | |
|  | | | | | | |  | | | | | |
| Urogenital or prostate tumour | | | | | | | Sequence 9 and 10 are mandatory (tT2 TSE and sT2 TSE) | | | | | |
|  | | | | | | |  | | | | | |
| LOCATION TUMOUR | | CHEST | | | First visit | | DO NOT ACQUIRE sequences 9 and 10 | | | | | |
|  | | | | | Follow-up | | DO NOT ACQUIRE sequences 9, 10 and 11 | | | | | |
|  | | UPPER ABDOMEN | | | First visit | | DO NOT ACQUIRE sequences 9 and 10 | | | | | |
|  | | | | | Follow-up | | DO NOT ACQUIRE sequences 9, 10 and 1 | | | | | |
|  | | PELVIC | | | First visit | | ALL SEQUENCES | | | | | |
|  | | | | | Follow-up | | SELECTION OF SEQUENCE 7, 8, 9 and 10.; NOT sequence 11 | | | | | |
|  | | | | | | | | | | | | |
| **PREPARATION** | | | | | | | | | | | | |
| Coil | | Posterior and anterior | | | | | | | | | | |
| Contrast | | Gadolinium (for instance, Gadovist 0.1 mL per kg body weight) | | | | | | | | | | |
|  | | | | | | | | | | | | |
| **WITH ANESTHESIA (SMALL CHILD)** | | | | | | | | | | | | |
|  | Sequence | | Technique | Orientation | | FOV [mm] | | Slices | Thickness [mm] | Voxel size [mm] | TE [ms] | TR [ms] |
| **1** | SURVEY | | FFE | MST | |  | |  |  |  |  |  |
| **2** | 3-D T2 TSE with pearbelt | | TSE | COR | | 400 x 353 | | 139 | 1.15 | 1.15 x 1.15 | 90 | shortest 455 |
| **3** | T1 THRIVE 1 | | TSE | TRA | | 380 x 331 | | 87 | 3.0 | 1.25 x 1.24 | shortest 2.9 | shortest 6 |
| **4** | T1 THRIVE 2 | | TSE | TRA | | 380 x 331 | | 87 | 3.0 | 1.25 x 1.24 | shortest 2.9 | shortest 6 |
| **5** | DWI (b=0; 100; 500; 1,000 s/mm2) and ADC map 1 | | EPI | TRA | | 380 x 332 | | 26 | 5.0 | 2.81 x 3.5 | shortest 78 | 4,000-5,000 |
| **6** | DWI (b=0; 100; 500; 1.000 s/mm2) and ADC map 2 | | EPI | TRA | | 380 x 332 | | 26 | 5.0 | 2.81 x 3.5 | shortest 78 | 4,000-5,000 |
| **7** | T1 TSE | | TSE | TRA | | 250 x 250 | | 65 | 4.0 | 0.7 x 0.8 | 8 | 400 / 600 |
| **8** | T2 MV xd FS | | TSE | TRA | | 300 x 300 | | 50 | 4.0 | 1 x 1 | 100 | 2,227 |
| **9** | T2 TSE* | | TSE | SAG | | 250 x 201 | | 39 | 4.0 | 0.65 x 0.92 | 100 | shortest 4,029 |
| **10** | T2 TSE* | | TSE | TRA | | 250 x 250 | | 65 | 4.0 | 0.75 x 0.95 | 100 | shortest 6,671 |
| **11** | T1 IN and OUT of phase* | | TSE | TRA | | 300 x 246 | | 25 | 5.0 | 1.67 x 2.09 | TE1: 2.3/ TE2: 4.3 | shortest 6 |
| **12** | Post-Gd T1 THRIVE 1^#^ | | TFE | TRA | | 380 x 331 | | 87 | 3 | 1.25 x 1.24 | shortest 2.9 | shortest 6 |
| **13** | Post-Gd T1 THRIVE 2^#^ | | TFE | TRA | | 380 x 331 | | 87 | 3.0 | 1.25 x 1.24 | shortest 2.9 | shortest 6 |
|  | *optional  ^#^ Post-Gd scans should be performed with fat saturation / water excitation. | | | | | | | |  |  |  |  |
|  | | | | | | | | | | | | |
| **WITHOUT ANESTHESIA (LARGE CHILD)** | | | | | | | | | | | | |
|  | Sequence | | Technique | Orientation | | FOV [mm] | | Slices | Thickness [mm] | Voxel size [mm] | TE [ms] | TR [ms] |
| **1** | SURVEY | | FFE | MST | |  | |  |  |  |  |  |
| **2** | T2 MV xd | | TSE | COR | | 450 x 450 | | 35 | 5.0 | 1 x 1 | 100 | shortest 2,457 |
| **3** | T1 THRIVE 1 | | TSE | TRA | | 380 x 299 | | 150 | 3.0 | 1.25 x 1.25 | shortest 2.9 | shortest 6 |
| **4** | T1 THRIVE 2 | | TSE | TRA | | 380 x 299 | | 150 | 3.0 | 1.25 x 1.25 | shortest 2.9 | shortest 6 |
| **5** | DWI (b=0; 100; 500; 1,000 s/mm2) and ADC map 1 | | EPI | TRA | | 380 x 380 | | 45 | 5.0 | 2.81 x 3.49 | shortest 78 | 4,000-5,000 |
| **6** | DWI (b=0; 100; 500; 1,000 s/mm2) and ADC map 2 | | EPI | TRA | | 380 x 380 | | 45 | 5.0 | 2.81 x 3.49 | shortest 78 | 4,000-5,000 |
| **7** | T1 TSE | | TSE | TRA | | 250 x 250 | | 65 | 4.0 | 0.75 x 0.8 | 8 | 400 / 600 |
| **8** | T2 MV xd FS | | TSE | TRA | | 300 x 300 | | 63 | 4.0 | 1 x 1 | shortest 57 | shortest 2,304 |
| **9** | T2 TSE* | | TSE | SAG | | 250 x 201 | | 39 | 4.0 | 0.65 x 0.92 | 100 | shortest 4,029 |
| **10** | T2 TSE* | | TSE | TRA | | 250 x 250 | | 65 | 4.0 | 0.75 x 0.95 | 100 | shortest 6,671 |
| **11** | T1 IN and OUT of phase* | | TSE | TRA | | 300 x 246 | | 25 | 5.0 | 1.67 x 2.09 | TE1:2.3/ TE2:4.3 | shortest 163 |
| **12** | Post-Gd T1 THRIVE 1^#^ | | TFE | TRA | | 380 x 299 | | 150 | 3 | 1.25 x 1.25 | shortest 2.9 | shortest 6 |
| **13** | Post-Gd T1 THRIVE 2^#^ | | TFE | TRA | | 380 x 299 | | 150 | 3.0 | 1.25 x 1.25 | shortest 2.9 | shortest 6 |
|  | * optional |  |  |  | |  | |  |  |  |  |  |

^#^ Post-Gd scans should be performed with fat saturation / water excitation.

**Extremities**

| **INDICATION** | | | | **PROTOCOL** | | | | | | |
| --- | --- | --- | --- | --- | --- | --- | --- | --- | --- | --- |
| Soft-tissue tumour arising from the extremities | | | | Before contrast agent administration: sequences 1 to 5 | | | | | | |
|  | | | | During contrast agent administration: sequence 6 | | | | | | |
|  | | | | After contrast agent administration: sequence 7 and 8 with SPIR | | | | | | |
| If possible mark swelling or scar | | | | | | | | | | |
|  | | | | | | | | | | |
| **PREPARATION** | | | | | | | | | | |
| Coil | | Posterior and anterior | | | | | | | | |
| Contrast agent | | Gadolinium (for instance, Gadovist 0.1 mL per kg body weight) | | | | | | | | |
|  |  |  |  |  |  |  |  |  |  |  |
|  | Sequence | | Technique | Orientation | FOV [mm] | Slices | Thickness [mm] | Voxel size [mm] | TE [ms] | TR [ms] |
| **1** | SURVEY | | FFE | MST |  |  |  |  |  |  |
| **2** | T1 TSE | | TSE | TRA | 200 x 200 | 29 | 4.0 | 0.7 x 0.86 | shortest 16 | 400 / 650 |
| **3** | T2 TSE mDIXON | | TSE | TRA | 200 x 200 | 29 | 4.0 | 0.8 x 0.95 | 80 | 3,500 / 5,500 |
| **4** | T1 | | TSE | SAG/COR | 300 x 300 | 31 | 3.0 | 0.95 x 0.95 | 15 | 540 / 650 |
| **5** | DWI (b=0; 100; 500; 1,000 s/mm2) and ADC map | | EPI | TRA | 200 x 200 | 40 | 4.0 | 2.5 x 2.5 | 72 | 4,000-5,000 |
| **6** | DCE (start < 20 s after infusion for fast temporal resolution) | | TFE | TRA | 200 x 200 | 8 | 5.0 | 1.17 x 2.94 | 1.3 | 5.4 |
| **7** | Post-Gd T1 TSE SPIR^#^ | | TSE | SAG/COR | 300 x 300 | 36 | 3.0 | 0.95 x 0.9 | 15 | 450 / 650 |
| **8** | Post-Gd T1 TSE SPIR^#^ | | TSE | TRA | 200 x 200 | 29 | 4.0 | 0.85 x 0.9 | 20 | 400 / 650 |

^#^ Post-Gd scans should be performed with fat saturation / water excitation.

## Siemens 3-T rhabdomyosarcoma MR template

**Head and neck**

| **INDICATION** | | | | | | **PROTOCOL** | | | | |
| --- | --- | --- | --- | --- | --- | --- | --- | --- | --- | --- |
| Soft-tissue tumour in the head or neck | | | | | | Before contrast agent administration: sequences 1 to 6 | | | | |
|  | | | | | | DCE optional | | | | |
| If possible mark swelling or scar | | | | | |  | | | | |
|  | | | | | | | | | | |
| **PREPARATION** | | | | | | | | | | |
| Coil | | Head / neck | | | | | | | | |
| Contrast agent | | Gadolinium (for instance, Gadovist 0.1 mL per kg body weight) | | | | | | | | |
|  |  |  |  |  |  |  |  |  |  |  |
|  | Sequence | | Technique | Orientation | FOV [mm] | Slices | Thickness [mm] | Voxel size [mm] | TE [ms] | TR [ms] |
| **1** | SURVEY | | TFE | MST |  |  |  |  |  |  |
| **2** | T1 TSE | | TSE | COR | 180 x 180 | 33 | 3 | 0.45 x 0.5 | 15 | 450 / 700 |
| **3** | T1 TSE | | TSE | TRA | 180 x 180 | 35 | 3 | 0.6 x 0.66 | shortest 15 | 450 / 750 |
| **4** | T2 TSE | | TSE | COR | 180 x 180 | 33 | 3 | 0.5 x 0.5 | 80 | 2,500 / 6,000 |
| **5** | T2 FS | | MV | TRA | 330 x 330 | 64 | 3 | 0.8 x 0.8 | 96 | 3,000 / 4,500 |
| **7** | DWI (b=0; 100; 500; 1,000 s/mm2) and ADC map | | EPI | TRA | 230 x 196 | 41 | 3 | 2.0 x 2.0 | shortest 75 | 4,000-5,000 |
| **8** | Post-Gd T1 VIBE | | TFE | TRA | 190 x 190 | 320 | 0.9 | 0.5 x 0.8 | shortest 3.2 | shortest 6.6 |
| **9** | Post-Gd T1 TSE DIXON^#^ | | TSE | TRA | 200 x 200 | 35 | 3 | 0.6 x 0.78 | 14 | 450 / 650 |
| **10** | Post-Gd T1 TSE DIXON^#^ | | TSE | COR | 180 x 180 | 33 | 3 | 0.6 x 0.78 | 14 | 450 / 650 |

^#^ Post-Gd scans should be performed with fat saturation / water excitation.

**Chest and abdomen**

1.5 T recommended, see 1.5-T protocol.

**Extremities**

| **INDICATION** | | | | **PROTOCOL** | | | | | | |
| --- | --- | --- | --- | --- | --- | --- | --- | --- | --- | --- |
| Soft-tissue tumour in extremity | | | | Before contrast agent administration: sequences 1 to 4 | | | | | | |
|  | | | | During contrast agent administration: sequence 7 | | | | | | |
|  | | | | After contrast agent administration: repeat sequence 2 and 3 with SPIR (=sequence 8 and 9) | | | | | | |
| If possible mark swelling or scar | | | |  | | | | | | |
|  | | | | | | | | | | |
| **PREPARATION** | | | | | | | | | | |
| Coil | | Posterior and anterior | | | | | | | | |
| Contrast agent | | Gadolinium (for instance, Gadovist 0.1 mL per kg body weight) | | | | | | | | |
|  | | | | | | | | | | |
|  | Sequence | | Technique | Orientation | FOV [mm] | Slices | Thickness [mm] | Voxel size [mm] | TE [ms] | TR [ms] |
| **1** | SURVEY | | FFE | MST |  |  |  |  |  |  |
| **2** | T1 TSE | | TSE | TRA | 250 x 250 | 30 | 4 | 0.8 x 1.1 | 10 | 400 / 750 |
| **3** | T2 TSE mDIXON or T2 SPAIR | | TSE | TRA | 250 x 250 | 30 | 4 | 0.87 x 1.09 | 80 | shortest 2,236 |
| **4** | T1 TSE | | TSE | SAG/COR | 200 x 200 | 40 | 3 | 0.8 x 0.9 | 10 | 500 / 700 |
| **5** | DWI (b=0; 100; 500; 1,000 s/mm2) and ADC map | | EPI | TRA | 230 x 230 | 50 | 4 | 2.5 x 2.5 | shortest 54 | 4,000-5,000 |
| **6** | DCE (start < 20 s after infusion for fast temporal resolution) | | TFE | TRA | 300 x 150 | 8 | 5 | 1.17 x 2.54 | 1.5 | shortest 6.1 |
| **7** | Post-Gd T1 TSE SPAIR^#^ | | TSE | SAG/COR | 200 x 200 | 40 | 3 | 0.8 x 0.9 | 10 | 500 / 700 |
| **8** | Post-Gd T1 TSE SPAIR^#^ | | TSE | TRA | 250 x 250 | 30 | 4 | 0.8 x 1.1 | shortest 10 | 400 / 750 |

^#^ Post-Gd scans should be performed with fat saturation / water excitation.

## Siemens 1.5-T rhabdomyosarcoma MR template

**Head and neck**

| **INDICATION** | | | | | **PROTOCOL** | | | | |
| --- | --- | --- | --- | --- | --- | --- | --- | --- | --- |
| Soft-tissue tumour in the head or neck | | | | | Before contrast agent administration: sequences 1 to 6 | | | | |
|  | | | | | DCE optional | | | | |
| If possible mark swelling or scar | | | | |  | | | | |
|  | | | | | | | | | |
| **PREPARATION** | | | | | | | | | |
| Coil | | Head / neck | | | | | | | |
| Contrast agent | | Gadolinium (for instance, Gadovist 0.1 mL per kg body weight) | | | | | | | |
|  | | | | | | | | | |
|  | Sequence | Technique | Orientation | FOV [mm] | Slices | Thickness [mm] | Voxel size [mm] | TE [ms] | TR [ms] |
| **1** | SURVEY | FFE | MST |  |  |  |  |  |  |
| **2** | T1 TSE | TSE | COR | 180 x 180 | 33 | 3 | 0.6 x 0.74 | 14 | 400 / 650 |
| **3** | T1 TSE | TSE | TRA | 180 x 180 | 35 | 3 | 0.6 x 0.74 | 16 | 400 / 650 |
| **4** | T2 TSE | TSE | COR | 180 x 180 | 33 | 3 | 0.45 x 0.5 | 100 | 2,500 / 3,500 |
| **5** | T2 FS | MV | TRA | 330 x 330 | 64 | 3 | 1.0 x 1.0 | shortest 76 | shortest 6,063 |
| **6** | DWI (b=0; 100; 500; 1,000 s/mm2) and ADC map | EPI | TRA | 200 x 200 | 41 | 4 | 2.81 x 2.81 | shortest 71 | 4,000-5,000 |
| **7** | Post-Gd VIBE^#^ | TFE | TRA | 190 x 190 | 320 | 1 | 0.8 x 0.9 | shortest 4.5 | shortest 9.6 |
| **8** | Post-Gd T1 TSE DIXON^#^ | TSE | TRA | 180 x 180 | 35 | 3 | 0.7 x 0.92 | 14 | 400 / 700 |
| **9** | Post-Gd T1 TSE DIXON^#^ | TSE | COR | 180 x 180 | 33 | 3 | 0.7 x 0.94 | 14 | 400 / 700 |

^#^ Post-Gd scans should be performed with fat saturation / water excitation.

**Chest and abdomen**

| **INDICATION** | | | | | **PROTOCOL** | | | | | | |
| --- | --- | --- | --- | --- | --- | --- | --- | --- | --- | --- | --- |
| Soft-tissue tumour in chest or abdomen | | | | | Before contrast agent administration: sequences 1 to 11 | | | | | | |
|  | | | | | After contrast agent administration: repeat sequence 3 and 4 (=sequence 12 and 13) | | | | | | |
|  | | | | |  | | | | | | |
|  | | | | | Sequence 7 to 11 set around the mass | | | | | | |
|  | | | | |  | | | | | | |
| Urogenital or prostate tumour | | | | | Sequence 9 and 10 are mandatory (tT2 TSE and sT2 TSE) | | | | | | |
|  | | | | | | | | | | | |
| LOCATION TUMOUR | | CHEST | | | First visit | | DO NOT ACQUIRE sequence 9 and 10 | | | | |
|  | | | | | Follow-up | | DO NOT ACQUIRE sequence 9, 10 and 11 | | | | |
|  | | UPPER ABDOMEN | | | First visit | | DO NOT ACQUIRE sequence 9 and 10 | | | | |
|  | | | | | Follow-up | | DO NOT ACQUIRE sequence 9, 10 and 11 | | | | |
|  | | PELVIC | | | First visit | | ALL SEQUENCES | | | | |
|  | | | | | Follow-up | | SELECTION OF SEQUENCE 7, 8, 9 and 10. NOT 11 | | | | |
|  | | | | | | | | | | | |
| **PREPARATION** | | | | | | | | | | | |
| Coil | | Posterior and anterior | | | | | | | | | |
| Contrast agent | | Gadolinium (for instance, Gadovist 0.1 mL per kg body weight) | | | | | | | | | |
|  | | | | | | | | | |  | |
| **WITH ANESTHESIA (SMALL CHILD)** | | | | | | | | | | | |
|  | Sequence | | Technique | Orientation | | FOV [mm] | | Slices | Thickness [mm] | TE [ms] | TR [ms] |
| **1** | SURVEY | | FFE | MST | |  | |  |  |  |  |
| **2** | 3-D T2 TSE respiratory triggering | | TSE | COR | | 400 x 353 | | 139 | 1.15 | 90 | shortest 455 |
| **3** | T1 VIBE 1 | | TSE | TRA | | 380 x 331 | | 87 | 3 | shortest 2.9 | shortest 6 |
| **4** | T1 VIBE 2 | | TSE | TRA | | 380 x 331 | | 87 | 3 | shortest 2.9 | shortest 6 |
| **5** | DWI (b=0; 100; 500; 1,000 s/mm2) and ADC map 1 | | EPI | TRA | | 380 x 332 | | 26 | 5 | shortest 78 | 4,000-5,000 |
| **6** | DWI (b=0; 100; 500; 1,000 s/mm2) and ADC map 2 | | EPI | TRA | | 380 x 332 | | 26 | 5 | shortest 78 | 4,000-5,000 |
| **7** | T1 TSE | | TSE | TRA | | 250 x 250 | | 65 | 4 | 8 | 400 / 600 |
| **8** | T2 BLADE FS | | TSE | TRA | | 300 x 300 | | 50 | 4 | 100 | 2,227 |
| **9** | T2 TSE* | | TSE | SAG | | 250 x 201 | | 39 | 4 | 100 | shortest 4,029 |
| **10** | T2 TSE* | | TSE | TRA | | 250 x 250 | | 65 | 4 | 100 | shortest 6,671 |
| **11** | T1 IN and OUT of phase* | | TSE | TRA | | 300 x 246 | | 25 | 5 | TE1: 2.3/ TE2: 4.3 | shortest 6 |
| **12** | Post-Gd T1 VIBE 1^#^ | | TFE | TRA | | 380 x 331 | | 87 | 3 | shortest 2.9 | shortest 6 |
| **13** | Post-Gd T1 VIBE 2^#^ | | TFE | TRA | | 380 x 331 | | 87 | 3 | shortest 2.9 | shortest 6 |
| *optional | | | | | | | | | | | |
| **WITHOUT ANESTHESIA (LARGE CHILD)** | | | | | | | | | | | |
|  | Sequence | | Technique | Orientation | | FOV [mm] | | Slices | Thickness [mm] | TE [ms] | TR [ms] |
| **1** | SURVEY | | FFE | MST | |  | |  |  |  |  |
| **2** | T2 BLADE xd | | TSE | COR | | 450 x 450 | | 35 | 5 | 100 | shortest 2,457 |
| **3** | T1 VIBE 1 | | TSE | TRA | | 380 x 299 | | 150 | 3 | shortest 2.9 | shortest 6 |
| **4** | T1 VIBE 2 | | TSE | TRA | | 380 x 299 | | 150 | 3 | shortest 2.9 | shortest 6 |
| **5** | DWI (b=0; 100; 500; 1,000 s/mm2) and ADC map 1 | | EPI | TRA | | 380 x 380 | | 45 | 5 | shortest 78 | 4,000-5,000 |
| **6** | DWI (b=0; 100; 500; 1,000 s/mm2) and ADC map 2 | | EPI | TRA | | 380 x 380 | | 45 | 5 | shortest 78 | 4,000-5,000 |
| **7** | T1 TSE | | TSE | TRA | | 250 x 250 | | 65 | 4 | 8 | 400/600 |
| **8** | T2 BLADE FS | | TSE | TRA | | 300 x 300 | | 63 | 4 | shortest 57 | shortest 2,304 |
| **9** | T2 TSE* | | TSE | SAG | | 250 x 201 | | 39 | 4 | 100 | shortest 4,029 |
| **10** | T2 TSE* | | TSE | TRA | | 250 x 250 | | 65 | 4 | 100 | shortest 6,671 |
| **11** | T1 IN and OUT of phase* | | TSE | TRA | | 300 x 246 | | 25 | 5 | TE1: 2.3/ TE2: 4.3 | shortest 163 |
| **12** | Post-Gd T1 VIBE 1^#^ | | TFE | TRA | | 380 x 299 | | 150 | 3 | shortest 2.9 | shortest 6 |
| **13** | Post-Gd T1 VIBE 2^#^ | | TFE | TRA | | 380 x 299 | | 150 | 3 | shortest 2.9 | shortest 6 |

*optional

^#^Post-Gd scans should be performed with fat saturation / water excitation.

**Extremities**

| **INDICATION** | | | | **PROTOCOL** | | | | | | |
| --- | --- | --- | --- | --- | --- | --- | --- | --- | --- | --- |
| Soft-tissue tumour arising from the extremities | | | | Before contrast agent administration: sequences 1 to 5 | | | | | | |
|  | | | | During contrast agent administration: sequence 6 | | | | | | |
|  | | | | After contrast agent administration: sequence 7 and 8 with SPIR | | | | | | |
| If possible mark swelling or scar | | | |  | | | | | | |
|  | | | | | | | | | | |
| **PREPARATION** | | | | | | | | | | |
| Coil | | Posterior and anterior | | | | | | | | |
| Contrast agent | | Gadolinium (for instance, Gadovist 0.1 mL per kg body weight) | | | | | | | | |
|  | | | | | | | | | | |
|  | Sequence | | Technique | Orientation | FOV [mm] | Slices | Thickness [mm] | Voxel size [mm] | TE [ms] | TR [ms] |
| **1** | SURVEY | | FFE | MST |  |  |  |  |  |  |
| **2** | T1 TSE | | TSE | TRA | 200 x 200 | 29 | 4 | 0.7 x 0.86 | shortest 16 | 400 / 650 |
| **3** | T2 TSE DIXON or T2 SPAIR | | TSE | TRA | 200 x 200 | 29 | 4 | 0.8 x 0.95 | 80 | 3,500 / 5,500 |
| **4** | T1 | | TSE | SAG/COR | 300 x 300 | 31 | 3 | 0.95 x 0.95 | 15 | 540 / 650 |
| **5** | DWI (b=0; 100; 500; 1,000 s/mm2) and ADC map | | EPI | TRA | 200 x 200 | 40 | 4 | 2.5 x 2.5 | 72 | 4,000-5,000 |
| **6** | DCE (start < 20 s after infusion for fast temporal resolution) | | TFE | TRA | 200 x 200 | 8 | 5 | 1.17 x 2.94 | 1.3 | 5.4 |
| **7** | Post-Gd T1 TSE SPAIR^#^ | | TSE | SAG/COR | 300 x 300 | 36 | 3 | 0.95 x 0.9 | 15 | 450 / 650 |
| **8** | Post-Gd T1 TSE SPAIR^#^ | | TSE | TRA | 200 x 200 | 29 | 4 | 0.85 x 0.9 | 20 | 400 / 650 |

^#^Post-Gd scans should be performed with fat saturation / water excitation.

## GE 3-T rhabdomyosarcoma MR template

**Head and neck**

| **INDICATION** | | | | | **PROTOCOL** | | | | | | |
| --- | --- | --- | --- | --- | --- | --- | --- | --- | --- | --- | --- |
| Soft-tissue tumour in the head or neck | | | | | Before contrast agent administration: sequences 1 to 6 | | | | | | |
|  | | | | | DCE optional | | | | | | |
| If possible mark swelling or scar | | | | |  | | | | | | |
|  | | | | | | | | | | | |
| **PREPARATION** | | | | | | | | | | | |
| Coil | | | Head / neck | | | | | | | | |
| Contrast agent | | | Gadolinium (for instance, Gadovist 0.1 mL per kg body weight) | | | | | | | | |
|  | | | | | | | | | | | |
|  | Sequence | Technique | | Orientation | | FOV [mm] | Slices | Thickness [mm] | Voxel size [mm] | TE [ms] | TR [ms] |
| **1** | Localizer | FGRE | | MST | |  |  |  |  |  |  |
| **2** | T1 | FSE | | COR | | 180 x 180 | 33 | 3.0 | 0.45 x 0.5 | 15 | 450 / 700 |
| **3** | T1 | FSE | | TRA | | 180 x 180 | 35 | 3.0 | 0.6 x 0.66 | shortest 15 | 450 / 750 |
| **4** | T2 | FSE | | COR | | 180 x 180 | 33 | 3.0 | 0.5 x 0.5 | 80 | 2,500 / 6,000 |
| **5** | T2 Fat suppression | FSE | | TRA | | 330 x 330 | 64 | 3.0 | 0.8 x 0.8 | 96 | 3,000 / 4,500 |
| **6** | DWI (B=0; 100; 500; 1,000 s/mm2) and ADC map | SE EPI | | TRA | | 230 x 196 | 41 | 3.0 | 2.0 x 2.0 | shortest 75 | 4,000-5,000 |
| **7** | Post-Gd LAVA^#^ | LAVA (FGRE) | | TRA | | 190 x 190 | 320 | 0.9 | 0.5 x 0.8 | shortest 3.2 | shortest 6.6 |
| **8** | Post-Gd T1 IDEAL^#^ | FSE | | TRA | | 200 x 200 | 35 | 3.0 | 0.6 x 0.78 | 14 | 450 / 650 |
| **9** | Post-Gd T1 IDEAL^#^ | FSE | | COR | | 180 x 180 | 33 | 3.0 | 0.6 x 0.78 | 14 | 450 / 650 |

^#^Post-Gd scans should be performed with fat saturation / water excitation.

**Chest and abdomen**

1.5 T recommended, see 1.5-T protocol.

**Extremities**

| **INDICATION** | | | | | **PROTOCOL** | | | | | | |
| --- | --- | --- | --- | --- | --- | --- | --- | --- | --- | --- | --- |
| Soft-tissue tumour in extremity | | | | | Before contrast agent administration: sequences 1 to 4 | | | | | | |
|  | | | | | During contrast agent administration: sequence 7 | | | | | | |
|  | | | | | After contrast agent administration: sequence 8 and 9 with SPIR | | | | | | |
| If possible mark swelling or scar | | | | |  | | | | | | |
|  | | | | | | | | | | | |
| **PREPARATION** | | | | | | | | | | | |
| Coil | | Posterior and anterior | | | | | | | | | |
| Contrast agents | | Gadolinium (for instance, Gadovist 0.1 mL per kg body weight) | | | | | | | | | |
|  | | | | | | | | | | | |
|  | Sequence | | Technique | Orientation | | FOV [mm] | Slices | Thickness [mm] | Voxel size [mm] | TE [ms] | TR [ms] |
| **1** | Localizer | | FGRE | MST | |  |  |  |  |  |  |
| **2** | T1 | | FSE | TRA | | 250 x 250 | 30 | 4.0 | 0.8 x 1.1 | 10 | 400 / 750 |
| **3** | T2 IDEAL | | IDEAL FSE | TRA | | 250 x 250 | 30 | 4.0 | 0.87 x 1.09 | 80 | shortest 2,236 |
| **4** | T1 | | FSE | SAG/COR | | 200 x 200 | 40 | 3.0 | 0.8 x 0.9 | 10 | 500 / 700 |
| **5** | DWI (B=0; 100; 500; 1,000 s/mm2) and ADC map | | SE EPI | TRA | | 230 x 230 | 50 | 4.0 | 2.5 x 2.5 | shortest 54 | 4,000-5,000 |
| **6** | DCE LAVA | | LAVA( FGRE) | TRA | | 300 x 150 | 8 | 5.0 | 1.17 x 2.54 | 1.5 | shortest 6.1 |
| **7** | Post-Gd T1 FS^#^ | | FSE | SAG/COR | | 200 x 200 | 40 | 3.0 | 0.8 x 0.9 | 10 | 500 / 700 |
| **8** | Post-Gd T1 FS^#^ | | FSE | TRA | | 250 x 250 | 30 | 4.0 | 0.8 x 1.1 | shortest 10 | 400 / 750 |

^#^Post-Gd scans should be performed with fat saturation / water excitation.

## GE 1.5-T rhabdomyosarcoma MR template

**Head and neck**

| **INDICATION** | | | | | | | **PROTOCOL** | | | |
| --- | --- | --- | --- | --- | --- | --- | --- | --- | --- | --- |
| Soft-tissue tumour in the head or neck | | | | | | | Before contrast agent administration: sequences 1 to 6 | | | |
|  | | | | | | | DCE optional | | | |
| If possible mark swelling or scar | | | | | | | | | | |
|  | | | | | | | | | | |
| **PREPARATION** | | | | | | | | | | |
| Coil | | Head / neck | | | | | | | | |
| Contrast agent | | Gadolinium (for instance, Gadovist 0.1 mL per kg body weight) | | | | | | | | |
|  | | | | | | | | | | |
|  | Sequence | | Technique | Orientation | FOV [mm] | Slices | Thickness [mm] | Voxel size [mm] | TE [ms] | TR [ms] |
| **1** | Localizer | | FGRE | MST |  |  |  |  |  |  |
| **2** | T1 | | FSE | COR | 180 x 180 | 33 | 3.0 | 0.6 x 0.74 | 14 | 400 / 650 |
| **3** | T1 | | FSE | TRA | 180 x 180 | 35 | 3.0 | 0.6 x 0.74 | 16 | 400 / 650 |
| **4** | T2 | | FSE | COR | 180 x 180 | 33 | 3.0 | 0.45 x 0.5 | 100 | 2,500 / 3,500 |
| **5** | T2 FS | | Propeller FSE | TRA | 330 x 330 | 64 | 3.0 | 1.0 x 1.0 | shortest 76 | shortest 6,063 |
| **6** | DWI (b=0; 100; 500; 1,000 s/mm2) and ADC map | | SE EPI | TRA | 200 x 200 | 41 | 4.0 | 2.81 x 2.81 | shortest 71 | 4,000-5,000 |
| **7** | Post-Gd LAVA^#^ | | LAVA FGRE | TRA | 190 x 190 | 320 | 1.0 | 0.8 x 0.9 | shortest 4.5 | shortest 9.6 |
| **8** | Post-Gd T1 IDEAL^#^ | | IDEAL FSE | TRA | 180 x 180 | 35 | 3.0 | 0.7 x 0.92 | 14 | 400 / 700 |
| **9** | Post-Gd T1 IDEAL^#^ | | IDEAL FSE | COR | 180 x 180 | 33 | 3.0 | 0.7 x 0.94 | 14 | 400 / 700 |

^#^Post-Gd scans should be performed with fat saturation / water excitation.

**Chest and abdomen**

| **INDICATION** | | | | | | | | **PROTOCOL** | | | | | |  |
| --- | --- | --- | --- | --- | --- | --- | --- | --- | --- | --- | --- | --- | --- | --- |
| Soft-tissue tumour in chest or abdomen | | | | | | | | Before contrast agent administration: sequence 1 to 11 | | | | | |  |
|  | | | | | | | | After contrast agent administration: repeat Sequence 3 and 4 (=sequence 12 and 13) | | | | | |  |
|  | | | | | | | |  | | | | | |  |
|  | | | | | | | | Sequence 7 to 11 set around the mass | | | | | |  |
|  | | | | | | | |  | | | | | |  |
| Urogenital or prostate tumour | | | | | | | | Sequence 9 and 10 are mandatory (tT2 TSE and sT2 TSE) | | | | | |  |
|  | | | | | | | |  | | | | | |  |
| LOCATION TUMOUR | | CHEST | | | First visit | | | DO NOT ACQUIRE sequence 9 and 10 | | | | | |  |
|  | | | | | Follow-up | | | DO NOT ACQUIRE sequence 9, 10 and 11 | | | | | |  |
|  | | UPPER ABDOMEN | | | First visit | | | DO NOT ACQUIRE sequence 9 and 10 | | | | | |  |
|  | | | | | Follow-up | | | DO NOT ACQUIRE sequence 9, 10 and 11 | | | | | |  |
|  | | PELVIC | | | First visit | | | ALL SEQUENCES | | | | | |  |
|  | | | | | Follow-up | | | SELECTION OF SEQUENCE 7, 8, 9 and 10. NOT 11 | | | | | |  |
|  | | | | | | | | | | | | | |  |
| **PREPARATION** | | | | | | | | | | | | | |  |
| Coil | | Posterior and anterior | | | | | | | | | | | |  |
| Contrast | | Gadolinium (for instance, Gadovist 0.1 mL per kg body weight) | | | | | | | | | | | |  |
|  | | | | | | | | | | | | | |  |
| **WITH ANESTHESIA (SMALL CHILD)** | | | | | | | | | | | | | |  |
|  | Sequence | | Technique | Orientation | | | FOV [mm] | | Slices | Thickness [mm] | Voxel size [mm] | TE [ms] | TR [ms] |  |
| **1** | Localizer | | FGRE | MST | | |  | |  |  |  |  |  |  |
| **2** | 3-D T2 CUBE RT | | FSE | COR | | | 400 x 353 | | 139 | 1.15 | 1.15 x 1.15 | 90 | shortest 455 |  |
| **3** | LAVA | | LAVA FGRE | TRA | | | 380 x 331 | | 87 | 3.0 | 1.25 x 1.24 | shortest 2.9 | shortest 6 |  |
| **4** | LAVA | | LAVA FGRE | TRA | | | 380 x 331 | | 87 | 3.0 | 1.25 x 1.24 | shortest 2.9 | shortest 6 |  |
| **5** | DWI (b=0; 100; 500; 1,000 s/mm2) and ADC map 1 | | SE EPI | TRA | | | 380 x 332 | | 26 | 5.0 | 2.81 x 3.5 | shortest 78 | 4,000-5,000 |  |
| **6** | DWI (b=0; 100; 500; 1,000 s/mm2) and ADC map 2 | | SE EPI | TRA | | | 380 x 332 | | 26 | 5.0 | 2.81 x 3.5 | shortest 78 | 4,000-5,000 |  |
| **7** | T1 | | FSE | TRA | | | 250 x 250 | | 65 | 4.0 | 0.7 x 0.8 | 8 | 400 / 600 |  |
| **8** | T2 propeller FS | | Propeller FSE | TRA | | | 300 x 300 | | 50 | 4.0 | 1 x 1 | 100 | 2,227 |  |
| **9** | T2 | | FSE | SAG | | | 250 x 201 | | 39 | 4.0 | 0.65 x 0.92 | 100 | shortest 4,029 |  |
| **10** | T2 | | FSE | TRA | | | 250 x 250 | | 65 | 4.0 | 0.75 x 0.95 | 100 | shortest 6,671 |  |
| **11** | LAVA Flex | | LAVA FGRE | TRA | | | 300 x 246 | | 25 | 5.0 | 1.67 x 2.09 | TE1: 2.3/ TE2: 4.3 | shortest 6 |  |
| **12** | Post-Gd LAVA^#^ | | LAVA FGRE | TRA | | | 380 x 331 | | 87 | 3 | 1.25 x 1.24 | shortest 2.9 | shortest 6 |  |
| **13** | Post-Gd LAVA^#^ | | LAVA FGRE | TRA | | | 380 x 331 | | 87 | 3.0 | 1.25 x 1.24 | shortest 2.9 | shortest 6 |  |
|  | * optional  ^#^Post-Gd scans should be performed with fat saturation / water excitation. | | | | | | | | | | | | |  |
| **WITHOUT ANESTHESIA (LARGER CHILD)** | | | | | | | | | | | | | |  |
|  | Sequence | | Technique | Orientation | | | FOV [mm] | | Slices | Thickness [mm] | Voxel size [mm] | TE [ms] | TR [ms] |  |
| **1** | Localizer | | FGRE | MST | | |  | |  |  |  |  |  |  |
| **2** | T2 propeller | | Propeller FSE | COR | | | 450 x 450 | | 35 | 5.0 | 1 x 1 | 100 | shortest 2,457 |  |
| **3** | LAVA | | LAVA FGRE | TRA | | | 380 x 299 | | 150 | 3.0 | 1.25 x 1.25 | shortest 2.9 | shortest 6 |  |
| **4** | LAVA | | LAVA FGRE | TRA | | | 380 x 299 | | 150 | 3.0 | 1.25 x 1.25 | shortest 2.9 | shortest 6 |  |
| **5** | DWI (b=0; 100; 500; 1,000 s/mm2) and ADC map 1 | | SE EPI | TRA | | | 380 x 380 | | 45 | 5.0 | 2.81 x 3.49 | shortest 78 | 4,000-5,000 |  |
| **6** | DWI (b=0; 100; 500; 1,000 s/mm2) and ADC map 2 | | SE EPI | TRA | | | 380 x 380 | | 45 | 5.0 | 2.81 x 3.49 | shortest 78 | 4,000-5,000 |  |
| **7** | T1 | | FSE | TRA | | | 250 x 250 | | 65 | 4.0 | 0.75 x 0.8 | 8 | 400 / 600 |  |
| **8** | T2 Propeller FS | | Propeller FSE | TRA | | | 300 x 300 | | 63 | 4.0 | 1 x 1 | shortest 57 | shortest 2,304 |  |
| **9** | T2 | | FSE | SAG | | | 250 x 201 | | 39 | 4.0 | 0.65 x 0.92 | 100 | shortest 4,029 |  |
| **10** | T2 | | FSE | TRA | | | 250 x 250 | | 65 | 4.0 | 0.75 x 0.95 | 100 | shortest 6,671 |  |
| **11** | LAVA Flex | | Flex FGRE | TRA | | | 300 x 246 | | 25 | 5.0 | 1.67 x 2.09 | TE1: 2.3/ TE2: 4.3 | shortest 163 |  |
| **12** | Post-Gd LAVA^#^ | | LAVA FGRE | TRA | | | 380 x 299 | | 150 | 3 | 1.25 x 1.25 | shortest 2.9 | shortest 6 |  |
| **13** | Post-Gd LAVA^#^ | | LAVA FGRE | TRA | | | 380 x 299 | | 150 | 3.0 | 1.25 x 1.25 | shortest 2.9 | shortest 6 |  |
|  | *optional | | | | |  | | | | | | | | |

^#^Post-Gd scans should be performed with fat saturation / water excitation.

**Extremities**

| **INDICATION** | | | | **PROTOCOL** | | | | | | |
| --- | --- | --- | --- | --- | --- | --- | --- | --- | --- | --- |
| Soft-tissue tumour arising from the extremities | | | | Before contrast agent administration: sequences 1 to 4 | | | | | | |
|  | | | | During contrast agent administration: sequence 6 | | | | | | |
|  | | | | After contrast agent administration: sequence 7 and 8 with SPIR | | | | | | |
| If possible mark swelling or scar | | | |  | | | | | | |
|  | | | | | | | | | | |
| **PREPARATION** | | | | | | | | | | |
| Coil | | Posterior and anterior | | | | | | | | |
| Contrast agent | | Gadolinium (for instance, Gadovist 0.1 mL per kg body weight) | | | | | | | | |
|  | | | | | | | | | | |
|  | Sequence | Technique | Orientation | | FOV [mm] | Slices | Thickness [mm] | Voxel size [mm] | TE [ms] | TR [ms] |
| **1** | Localizer | FGRE | MST | |  |  |  |  |  |  |
| **2** | T1 | FSE | TRA | | 200 x 200 | 29 | 4.0 | 0.7 x 0.86 | shortest 16 | 400 / 650 |
| **3** | T2 IDEAL | IDEAL FSE | TRA | | 200 x 200 | 29 | 4.0 | 0.8 x 0.95 | 80 | 3,500 / 5,500 |
| **4** | T1 | FSE | SAG/COR | | 300 x 300 | 31 | 3.0 | 0.95 x 0.95 | 15 | 540 / 650 |
| **5** | DWI (B=0; 100; 500; 1,000 S/MM2) and ADC map | SE EPI | TRA | | 200 x 200 | 40 | 4.0 | 2.5 x 2.5 | 72 | 4,000-5,000 |
| **6** | DCE LAVA | LAVA FGRE | TRA | | 200 x 200 | 8 | 5.0 | 1.17 x 2.94 | 1.3 | 5.4 |
| **7** | Post-Gd T1 FS^#^ | FSE | SAG/COR | | 300 x 300 | 36 | 3.0 | 0.95 x 0.9 | 15 | 450 / 650 |
| **8** | Post-Gd T1 FS^#^ | FSE | TRA | | 200 x 200 | 29 | 4.0 | 0.85 x 0.9 | 20 | 400 / 650 |

^#^Post-Gd scans should be performed with fat saturation / water excitation.
