# Supplementary material for: European guideline for imaging in paediatric and adolescent rhabdomyosarcoma — joint statement by the European Paediatric Soft Tissue Sarcoma Study Group, the Cooperative Weichteilsarkom Studiengruppe and the Oncology Task Force of the European Society of Paediatric Radiology
Source: Pediatr Radiol. 2021 Jun 17;51(10):1940–51. doi: 10.1007/s00247-021-05081-0 (PMC8426307; doi:10.1007/s00247-021-05081-0)
Supplement: Supplementary file 2 — (DOCX 15 kb) [file 247_2021_5081_MOESM2_ESM.docx]

# Appendix B

# Standard reporting templates

# B.1 Standard template for reporting chest CT in paediatric and adolescent rhabdomyosarcoma

**Technique:**

CT *<with/without>* intravenous contrast.

<*..>* mm axial slices.

**Interpretation (this is an example and not mandatory):**

The examination is compared with scan dated *<insert date>*.

Pathologic osseous or soft-tissue processes *<are – are not>* present. {If present describe}

Enlarged axillary lymph nodes *<are – are not>* present. {If present describe and measure minimal axis}

Pleural fluid or thickening *<is – is not>* present. {If present describe amount and side}

Pulmonary parenchymal nodules *<are – are not>* present. {If present describe location and measure maximum axis}

The tracheobronchial tree *<is – is not>* normal. {If abnormal describe}

A mediastinal mass *<is – is not>* present.

Enlarged mediastinal lymph nodes *<are – are not>* present. {If present describe location and measure maximum axis}

**Conclusion (the conclusion should at least contain these parameters):**

There are

*– no nodules*

*– indeterminate nodules {i.e. no more than four nodules of less than 5 mm or one nodule measuring between 5 mm and less than 10 mm}*

*– pulmonary metastases {i.e. more than four nodules of less than 5 mm or more than one nodule measuring between 5 mm and less than 10 mm or more than one nodule measuring more than 10 mm}>*. {if pulmonary metastases are present specifically report the diameter and lobar location of the two largest metastases}

{In case of follow-up study} Compared with the previous chest CT there is *< regression of disease – no change – progression of disease>*.

# B.2 Standard template for reporting MRI in paediatric and adolescent rhabdomyosarcoma

### MRI – HEAD AND NECK

**Technique (this is an example and not mandatory):**

<volume> mL of <contrast agent> was administered per protocol.

The following sequences were obtained: <name sequences applied>

**Interpretation (this is an example and not mandatory):**

**Primary tumour (T-stage):**

{Short description of tumour, location, relation with other tissues and extension}

1. Location primary tumour:
   1. Origin primary tumour and specify extension in different directions {location according to space description}
   2. Tissue origin of tumour {cutis, subcutis, muscle, fascia, etc.}
   3. Side {right/left/midline}
2. Local extension primary tumour:
   1. Extension of tumour in different directions {name spaces}
   2. Vascular encasement of the carotid artery (No/Yes} {if Yes, name vessels}
   3. Perineural extension {No/Yes} {if Yes, describe}
   4. Leptomeningeal/dural involvement {No/Yes < or > than 5 mm} {If Yes, describe}
   5. Intracranial extension {Yes/No} {If Yes, describe}
   6. Bone involvement or bone erosion (No/Yes} {if Yes, describe}
3. Measurements:
   1. Three dimensions (axial plane 2 dimensions; cor/sag 1 dimension)
   2. Volume (three dimension * 0,52)
4. Characteristics:
   1. Signal characteristics {T1, T2, T2 fat sat}
   2. Enhancement {homogeneous, heterogeneous, with/without necrosis}
   3. DWI {with/without diffusion restriction}
5. Other findings of the primary tumour:
   1. Perilesional oedema in surrounding soft tissue {No/Yes}
   2. Multifocality {No single lesion / Yes multifocal lesion}
   3. (Lymphangitic) skin involvement {Yes/No} {If Yes, describe}

**Lymph node involvement (N-stage):**

Suspicious (defined as short axis 5-15 mm) or pathologic lymph nodes retropharyngeal and neck regions (defined as lack of fatty centre and maximum short axis >15 mm) {No/Yes} {if Yes, describe number, maximum short axis diameter and location}

**Metastases (M-stage):**

Other suspicious lesions {No/Yes} {if Yes, describe number, size and location}

**Other important findings:**

-{No/Yes} {if Yes, describe}

**Conclusion (the conclusion should at least address the following):**

First MRI examination:

Primary tumour: Site of origin and extension in different {spaces}.

Therefore the tumour is in a {parameningeal/non-parameningeal/orbital} site.

Follow-up Study:

Compared with the study dated <DATE> there is <Complete Response – Partial Response (<NUMBER> %) – Stable Disease – Progressive Disease.

{In case of follow-up study} Compared with the study dated <DATE> there is <Complete Response – Partial Response (<NUMBER> %) – Stable Disease – Progressive Disease.

### MRI – CHEST AND ABDOMEN

**Technique (this is an example and not mandatory):**

<volume> mL of <contrast agent> was administered per protocol.
The following sequences were obtained: <name sequences applied>

**Interpretation (this is an example and not mandatory):
Primary tumour:**{Report size, in three dimensions, location and extension}

**Chest:**

Pulmonary parenchymal nodules *<are – are not>* present. {If present they should be measured on chest CT}

Enlarged axillary lymph nodes *<are – are not>* present. {If present describe and measure minimal axis}

Pleural fluid or thickening *<is – is not>* present. {If present describe amount and side}

**Mediastinum:**
A pathological mediastinal mass *<is – is not>* present.
Enlarged mediastinal lymph nodes *<are – are not>* present. {If present describe location and measure maximum axis}

**Liver:**The liver measures <length> cm. and is <small – normal – large> for age.
The liver shows <no metastases – metastases> { If present describe and measure the two largest lesions}
The intra and extra hepatic bile ducts are <non-dilated – dilated>.
The portal vein is <non-patent – patent>.
The hepatic veins are <non-patent – patent>.

**Right Kidney:**
The kidney measures <length> cm. and is <small – normal – large> for age.
The kidney has <normal parenchyma – abnormal parenchyma> {If abnormal describe}.
The renal collecting system is <non-dilated – dilated>.
The ureter is <non-dilated – dilated>.
There is a <normal – enlarged> adrenal gland. {if abnormal record maximum diameter}

**Left Kidney:**The kidney measures <length> cm. and is <small – normal – large> for age.
The kidney has <normal parenchyma – abnormal parenchyma> {If abnormal describe}.
The renal collecting system is <non-dilated – dilated>.
The ureter is <non-dilated – dilated>.
There is a <normal – enlarged> adrenal gland. {if abnormal record maximum diameter}

**Spleen:**
The spleen measures <length> cm. and is <small – normal – large> for age.
The spleen has a <abnormal – normal> parenchymal aspect. {If abnormal describe}.

**Pancreas:**
The pancreas has a <abnormal – normal> parenchymal aspect. {If abnormal describe}.
The pancreatic duct is <non-dilated – dilated>.

**Lymph nodes:**{report maximum small axis diameter of pathological lymph nodes, i.e. defined as lack of fatty centre and a minimum short axis diameter of 15 mm, measure the two largest nodes}

**Other lesions:**{The following lesions cannot be measured but should be reported: small lesions (longest diameter <10 mm or pathological lymph nodes with >10 to <15 mm short axis) as well as truly non-measurable lesions. Lesions considered truly non-measurable include: ascites, pleural or pericardial effusion, inflammatory breast disease, lymphangitic involvement of skin or lung }

**Conclusion (the conclusion should at least contain these parameters):**First MRI study:

{In case of follow-up study} Compared to the study dated <DATE> there is <Complete Response – Partial Response (<NUMBER> %) – Stable Disease – Progressive Disease.

### MRI – EXTREMITIES

**Technique (this is an example and not mandatory):**

<volume> mL of <contrast agent> was administered per protocol.
The following sequences were obtained: <name sequences applied>

**Interpretation (this is an example and not mandatory):

Primary tumour (T-stage):**

{Short description of tumour, location, relation with other tissues and extension}

1. Location:

- Extremity (Upper arm/Forearm, etc.}
- Side {Right/Left}
- Origin of the tumour {cutis, subcutis, muscle, fascia, etc.}
- Anatomical compartments involved {anterior, posterior, lateral, etc.}

2. Measurements:

- Three dimensions (axial plane 2 dimensions; cor/sag 1 dimension)
- Volume (three dimension * 0,52)

3. Characteristics:

- Signal characteristics {T1, T2, T2 fat sat}
- Enhancement {homogeneous, heterogeneous, with/without necrosis}
- DWI {with/without diffusion restriction}

4. Local extension primary tumour:

- Perilesional oedema in surrounding soft tissue {No/Yes}
- Multifocality {No single lesion / Yes multifocal lesion}
- Bone involvement (No/Yes} {if Yes, describe}
- Joint involvement {No/Yes} {if Yes, name joint}
- Neurovascular bundle involvement (No/Yes} {if Yes, name NVBs}

**Lymph node involvement (N-stage):**

Suspected (defined by RECIST 1.1) or pathologic lymph nodes (defined as lack of fatty centre and maximum short axis >10 mm) {No/Yes} {if Yes, describe number, maximum short axis diameter and location}

**Metastases (M-stage):**

Other suspect lesions {No/Yes} {if Yes, describe number, size and location}

**Other important findings:**

-{No/Yes} {if Yes, describe}

**Conclusion (the conclusion should at least contain these parameters):**First MRI study:

{In case of a follow-up study} Compared with the study dated <DATE> there is <Complete Response – Partial Response (<NUMBER> %) – Stable Disease – Progressive Disease.

# B.3 Standard template for reporting FDG PET in paediatric and adolescent rhabdomyosarcoma

FDG activity at time of calibration:

Time of administration:

Time of start of acquisition:

Serum glucose:

Preparation: *(propranolol use and dose yes/no)*

Scan range: Should be whole body, if additional scan is performed *(e.g. separate head & neck acquisition*) this should be notified.

**Primary tumour:**

● Location

● FDG uptake* (visual assessment) using the Deauville score) + semi-quantitative (SUVmax)

● Maximum diameter

● Metabolic tumour volume (MTV)

**Regional lymph node metastases:**

● Location

● FDG uptake* (visual assessment using the Deauville score) + semi-quantitative (SUVmax)

● Number of metastases

● Size of 2 largest metastases

**Distant lymph node metastases:**

● Location

● FDG uptake* (visual assessment using the Deauville score) + semi-quantitative (SUVmax)

● Number of metastases

● Size of 2 largest metastases

**Distant metastasis yes / no:**

● Location

● FDG uptake* (visual assessment using the Deauville score) + semi-quantitative (SUVmax)

● Number of metastases

● Size 2 largest pathological nodes

**Miscellaneous / other findings:**

e.g., diffuse bone marrow uptake, infection, pleural fluid / ascites etc.

**FDG uptake interpretation and conclusion:**

Describe the metabolic activity of the primary tumour and/or pathological lymph nodes and/or distant metastases according to lexicon*, based on frequently Deauville score for malignant lymphoma, where moderate to intense uptake is considered metabolically active tumour tissue.

*Lexicon FDG uptake:

- normal / low score 1 / 2 uptake below or equal to blood pool activity)

- mild score 3 uptake > bloodpool activity, but < liver activity)

- moderate score 4 uptake ≥ liver activity)

- intense score 5 uptake >>> liver uptake)
